# Editorial: The role of lipids in relation to preventing inflammation and chronic diseases

**DOI:** 10.3389/fnut.2026.1944663

**Published:** 2026-08-12

**Authors:** Ioannis Zabetakis, Ian Megson, Alexandros Tsoupras, Constantina Nasopoulou, Ronan Lordan

**Affiliations:** 1 Department of Chemical Sciences, University of Limerick, Castletroy, Limerick, Ireland; 2 Bernal Institute, University of Limerick, Castletroy, Limerick, Ireland; 3 Health Research Institute, University of Limerick, Castletroy, Limerick, Ireland; 4 School of Chemistry, University of St Andrews, St Andrews, United Kingdom; 5 Hephaestus Laboratory, Faculty of Sciences, School of Chemistry, Democritus University of Thrace, Kavala, Greece; 6 Laboratory of Food Chemistry-Technology and Quality of Animal Origin Food, Department of Food Science and Nutrition, School of Environment, University of the Aegean, Lemnos, Greece; 7 School of Pharmacy and Biomolecular Sciences, RCSI University of Medicine and Health Sciences, Dublin, Ireland; 8 FutureNeuro Research Ireland Centre for Translational Brain Science, RCSI University of Medicine and Health Sciences, Dublin, Ireland; 9 Tissue Engineering Research Group (TERG), RCSI University of Medicine and Health Sciences, Dublin, Ireland

**Keywords:** cardiovascular diseases, chronic kidney disease, high-density lipoprotein, inflammation, non-communicable diseases, nutrition, obesity, polyunsaturated fatty acids

Despite substantial advances in pharmaceutical treatments targeting inflammation and the chronic non-communicable diseases (NCDs) associated with it, these conditions continue to cause considerable morbidity and mortality worldwide. NCDs, including cardiovascular disease (CVD), cancer, obesity, type 2 diabetes mellitus (T2DM), metabolic syndrome, and stroke, remain the leading causes of ill health and premature death. Indeed, CVD alone is responsible for over 18 million deaths annually, while cancer and obesity claim 10 million and 4.7 million deaths, respectively. These figures highlight the profound societal and healthcare burden of chronic illness. In recent years, chronic low-grade systemic inflammation has been increasingly recognized as a key contributor to the development and progression of many non-communicable diseases (NCDs). Addressing the mechanisms underlying systemic inflammation may therefore offer important opportunities for the prevention and management of these conditions. In spite of therapeutic advances, treatments often show variable effectiveness, underscoring the need for a deeper understanding of the mechanisms of NCDs. Lipids have been long recognized as structural and energy-storing molecules; today bioactive lipids regulate inflammatory and immune responses through multiple signaling pathways. Studying the diverse roles of lipids may uncover novel pathways in NCDs and inform the development of more precise, personalized interventions. Nutritional strategies and especially personalized nutrition represents a promising avenue for disease prevention and management.

The goal of this Research Topic was multidimensional. There is a clear and urgent need to better understand which mechanisms are related to the onset and progression of NCDs and how different lipids play a role in the onset, progression, and amelioration of these diseases. Previous research on this topic has shown that specific lipid nutrients can play a pivotal role in inhibiting inflammation and therefore the development of chronic diseases. It is of high interest to look into the research investigating dietary or exogenous lipids or other nutrients affecting lipid metabolism or pathways that may have a positive impact on reducing inflammation. Thus, this Research Topic addressed major gaps in our academic and medical knowledge, which may inform new research avenues.

Several studies in this Research Topic converge on remnant cholesterol (RC) as an emerging biomarker across multiple chronic diseases. Li, Chen, et al. studied how cardiometabolic risk factors (CMRF) are related to remnant cholesterol (RC) and diabetes. Their study focused on two East Asian cohorts. They performed a study using data from a Chinese (*n* = 112,694) and Japanese (*n* = 12,489) cohorts, stratified by CMRF status. The association between RC and diabetes mellitus (DM) was assessed using Cox regression models, restricted cubic splines (RCS), extreme gradient boosting (XGboost), receiver operating characteristic (ROC) analysis, and mediation analysis. RC was significantly associated with DM only in individuals with CMRF in both cohorts, and the association was non-linear. Their study found also differences between the two cohorts: RC showed better predictive performance compared to LDL-C and total cholesterol in predicting DM risk among individuals with CMRF (AUC 0.606 and 0.647 in Chinese and Japanese cohorts, respectively). CMRF mediated 76.3 and 66.3% of the RC-DM association in the Chinese and Japanese cohorts. Their conclusion was that RC is non-linearly associated with the incidence of DM in individuals with CMRF, primarily promoting its onset through the presence of CMRF.

Emerging evidence also indicates that remnant cholesterol (RC) and inflammation play a crucial role in diabetic kidney disease (DKD) pathogenesis. Xie X. et al. investigated the association and diagnostic efficacy of remnant cholesterol inflammatory index (RCII), integrating RC and inflammatory markers, with DKD. They found both overall and non-linear positive correlations between the risk of DKD and both RC (*P* for overall < 0.001, *P* for non-linear = 0.049) and RCII (*P* for overall < 0.001, *P* for non-linear < 0.001). Also, machine learning models incorporating RCII and traditional risk factors demonstrated robust diagnostic efficacy, with extreme gradient boosting (XGBoost) achieving the highest AUC values in the testing set (AUC: 0.953). Given that RCII is a novel and promising biomarker for DKD risk, they suggested that its integration into diagnostic models may enhance early identification and personalized prevention strategies for DKD.

An independent study conducted by Xie R. et al. investigated whether RC can be a novel risk factor for osteoarthritis (OA). They carried out a large prospective cohort of middle-aged and older adults; where RC showed a positive, dose–response association with incident osteoarthritis that attenuated to near-null after adjustment for BMI. Mediation analysis indicated that approximately 84% of the total association operated via BMI, supporting adiposity as the principal pathway and suggesting limited BMI-independent effect of RC. Their results highlighted that RC is potential modifiable metabolic biomarker and underscore the interplay of dyslipidemia and obesity in OA pathogenesis, suggesting that RC management combined with weight control may offer an effective strategy for OA prevention.

In another study under the theme on how RC is implicated to a number of CD, Ren et al. studied the association of plasma remnant cholesterol with cognitive function in middle-aged and elderly Chinese adults with T2DM in a cross-sectional study. They recruited one thousand eight hundred seventeen participants aged 55 to 75 from communities in Beijing. Demographic information and daily dietary intakes were collected by self-designed questionnaire. Fasting venous blood was obtained for quantitative analysis of plasma lipid parameters. The Montreal Cognitive Assessment (MoCA) was used to assess cognitive function. They identified that an increase in plasma RC level is a potential risk factor for MCI. A plasma RC concentration below 0.578 mmol/L was associated with a decreased risk of MCI in middle-aged and older individuals with T2DM. In a similar pattern, a plasma RC concentration below 0.581 mmol/L may lower the risk of MCI in non-T2DM subjects. The daily consumption of vegetables and legumes could help reduce the concentration of RC.

Collectively, these studies demonstrate that RC is not only associated with CVD, but it is also associated with risk of T2DM, diabetic kidney disease, osteoarthritis, and cognitive decline. Collectively, these findings reinforce the concept that remnant lipoproteins are more than passive lipid carriers, instead acting as important mediators of cardiometabolic dysfunction and inflammation.

Staying with the theme of lipoproteins, the role of high-density lipoproteins (HDL) in NCDs was also an examined in this Research Topic. Geng et al. investigated the association between HDL subfractions and long-term major adverse cardiac and cerebrovascular events (MACCEs) in patients with acute myocardial infarction (AMI). During a median follow-up of 52.4 months, 132 MACCEs (10.7%) occurred. Patients in the highest HDL-3 tertile had a lower MACCEs incidence than those in the lowest tertile (6.7% vs. 16.4%, *p* < 0.001). Higher HDL-3 levels were associated with improved event-free survival (HR = 0.58, 95% CI: 0.37–0.91) and demonstrated discriminative ability for MACCE risk (AUC = 0.62; 95% CI: 0.57–0.67), whereas HDL-C and HDL-2b were not significant. The levels of HDL-3 levels were independently associated with a higher long-term risk of MACCEs in AMI patients. Thus, HDL-3 may represent a potential biomarker for residual cardiovascular risk stratification and a potential therapeutic target for improving post-infarction outcomes.

HDL may also play a role in non-CVD related events. Sun et al. studied the association between the non-HDL to HDL cholesterol ratio and non-alcoholic fatty liver disease (NAFLD) in Chinese adults with and without gout. The non-HDL cholesterol to HDL cholesterol ratio (NHHR) serves as a comprehensive lipid index. Therefore, their study aimed to the association between NHHR and the risk of NAFLD in patients with gout. Their analysis of quartile groups stratified by NHHR levels revealed an increased prevalence of NAFLD corresponding to higher NHHR levels. They addressed their research question using multifactorial logistic regression analysis and they established a significant association between NHHR and NAFLD, yielding an odds ratio (OR) of 1.242 [95% confidence interval (CI): 1.089–1.416, *p* = 0.001]. Their study identified a positive correlation between NHHR and the incidence of NAFLD in individuals with gout, suggesting that NHHR may serve as a reliable indicator of NAFLD within the gout patient.

The systematic review of Mustafa et al. aimed to present an updated evidence synthesis on the potential link between circulating Lp(a) concentrations and this prevalent hepatic disease in adults. Twenty-one observational studies were included in their meta-analysis (137,494 cases; 281,261 controls). A three-level meta-analysis resulted in a pooled mean difference of 1.40 mg/dL [95% confidence interval: −2.81, 5.61; *p* = 0.50], indicating no significant difference in circulating Lp(a) concentrations between patients with Metabolic dysfunction-associated steatotic liver disease (MASLD) or NAFLD or metabolic dysfunction-associated fatty liver disease (MAFLD) and controls. They highlighted the limited potential for circulating Lp(a) as a diagnostic/prognostic biomarker for MASLD has limited potential, although this biomarker could still be utilized to assess CVD risk in the context of steatotic liver disease.

While dyslipidemia is synonymous with CVD, Ruan et al. provided a retrospective cohort study on renal outcomes and predictive value of dyslipidemia in patients with IgA nephropathy. Even though dyslipidemia is common in chronic kidney disease (CKD), including immunoglobulin A (IgA) nephropathy (IgAN), and may be associated with renal prognosis; the value of dyslipidemia in IgAN remains insufficiently assessed. Their investigation focused on clinicopathological characteristics and renal outcomes in IgAN patients with dyslipidemia and evaluated the prognostic value of lipid abnormalities. IgAN patients with dyslipidemia exhibited more severe clinicopathological features. Tubular atrophy/interstitial fibrosis and arteriosclerosis/arteriolosclerosis were closely associated with dyslipidemia. They concluded that dyslipidemia not only indicates adverse renal outcomes but also serves as an independent prognostic predictor.

CVD can be studied using atherosclerosis index and the group of Li, Gu, et al. carried out a prospective cohort analysis from the China Health and Retirement Longitudinal Study in order to study whether Cumulative Atherosclerosis Index of Plasma (CumAIP) is related to new-onset diabetes in middle-aged and older adults. They used data from the China Health and Retirement Longitudinal Study (CHARLS) and they analyzed 10,395 diabetes-free participants at baseline (2011) with follow-ups in 2013, 2015, and 2018. Multivariable logistic regression was adjusted for sociodemographic (age, gender, education), lifestyle (smoking, alcohol, sleep, physical activity), and clinical factors (systolic and diastolic blood pressure, BMI, waist circumference). They concluded that CumAIP exposure independently predicts diabetes incidence in middle-to-older adults and this result highlighted its potential for clinical risk stratification.

Over the past years, there has been considerable research on the role of polyunsaturated fatty acids (PUFAs) and their role in the development and treatment of NCDs. In the review paper of Kouti et al. the role of PUFAs as a potential preventive and therapeutic intervention for MASLD and its progression to hepatocellular carcinoma was discussed. MASLD is currently the leading cause of chronic liver disease worldwide and a major cause of hepatocellular carcinoma (HCC), a cancer with poor prognosis. Considering the immense public health impact of MASLD and MASLD-HCC, preventive and more effective management strategies for these diseases are urgently needed. Their review discussed the role of PUFAs, more specifically n-3 and n-6, in MASLD and MASLD-HCC, by critically reviewing evidence from human clinical and observational studies, and experimental models.

In another study of PUFAs in health, Huang et al. examined whether omega-6/omega-3 fatty acid ratio (O6:O3) measured by nuclear magnetic resonance (NMR) spectroscopy can improve cardiovascular risk prediction. The plasma O6:O3 ratio, a marker of inflammatory balance, is a promising biomarker for risk stratification. Therefore, they aimed to evaluate if adding the O6:O3 ratio to the SCORE2 model improves the prediction of major adverse cardiovascular events (MACE). They compared the predictive performance of the SCORE2 model with and without the O6:O3 ratio in an independent validation cohort (*N* = 54,940) using Harrell's C-index, net reclassification improvement (NRI), and integrated discrimination improvement (IDI).They concluded that incorporating the plasma O6:O3 PUFA ratio can provide a modest but statistically significant improvement in 10-year MACE risk prediction with the SCORE2 algorithm. Also, the O6:O3 ratio, being a modifiable biomarker, may has the potential to refine risk stratification and guide personalized nutritional interventions.

The *trans-*fatty acids are another type of dietary fatty acids implicated in CVD development. Ye et al. provided some meaningful insights on the impact of diets high in *trans*-fatty acids (TFA) on CVD in adults older than 55 years. They studied global trends and health inequalities in CVD burden attributable to high TFA intake from 1990 to 2021 and project future patterns through 2036. Using data from the Global Burden of Disease (GBD) Study 2021, they analyzed age-standardized mortality rates (ASMR), disability-adjusted life years (ASDR), and inequality indicators across 204 countries and territories. Age-Period-Cohort (APC) models and Bayesian projections were applied to estimate future trends. While global progress in reducing TFA-related CVD burden is evident, persistent disparities and emerging risks in low-resource settings still exist and underscore the need for global elimination of industrial TFA, strengthened health systems, and targeted strategies to protect high-risk groups.

Ketone bodies derived from ketogenesis of fatty acids in the liver, are water-soluble molecules that are vital alternative energy sources when glucose availability is limited, which in recent years have been of interest for other biological effects. Dai et al. published a review paper with some novel insights on how ketone bodies (KBs) and their metabolism affect kidney diseases. They discussed the role of KBs as important energy fuels and their complex roles in the occurrence and development of diseases by regulating metabolism, inflammation, and multi-organ cellular crosstalk. They highlighted that high mortality and morbidity of kidney diseases (KDs) is an urgent global public health problem. The pathogenesis of KDs is complex, with metabolic disorders underlying. Ketone body metabolism has recently become a hot spot, and ketogenic diets (KDs), sodium-glucose cotransporter 2 inhibitors (SGLT2i) along with exogenous steatotic liver disease body supplements have drawn much attention for their role in treating obesity and type 2 diabetes. They suggested that it is essential to clarify the effects of KBs on kidney diseases to understand the occurrence and development of kidney diseases correctly and guide clinical treatment.

Zhao et al. presented a pharmacological and metabolomic exploration on how nutrients of red yeast rice extract (RYRE) can have an impact on liver health. They investigated the therapeutic mechanism of red yeast rice extract (Xuezhikang, XZK) in high-fat diet (HFD)-induced non-alcoholic fatty liver disease (NAFLD) through untargeted metabolomics analysis and experimental validation. They performed an untargeted metabolomics analysis based on UHPLC-QTOF/MS in order to identify differential metabolites in liver tissues. A NAFLD model was established in hamsters by HFD feeding. They found that XZK effectively ameliorated hepatic steatosis, dyslipidemia, and inflammation in HFD-induced NAFLD hamsters. The therapeutic effects were mediated through restoration of metabolic homeostasis and suppression of the JNK/AP-1/TNF-α signaling pathway.

Finally, although an acute condition, the impact of nutrition and NCDs on the outcomes of the viral infection requires further research. Bleffgen et al. addressed these aspects via an observational study on the micronutrient status and fatty acid profile of adults with SARS-CoV-2 infection. Micronutrient levels (vitamin D, selenium, zinc, magnesium, and iron) and lipid profiles between 139 SARS-CoV-2 -positive patients (62 hospitalized, 77 home care) and 314 healthy controls, were compared using dried blood spots. Differences by treatment setting (hospitalized vs. home care) as a proxy for disease severity were also examined. Patients with SARS-CoV-2 infection exhibited similar micronutrient levels but showed a significantly impaired lipid profile compared to healthy controls. Notably, there was a significant decrease in palmitic (*p*-value < 0.01) and stearic acid levels (*p*-value < 0.01) while a significant increase in omega-3 and omega-6 PUFAs, like AA (*p*-value < 0.01), DHA (*p*-value < 0.01), and EPA (*p*-value < 0.05) was observed. In the SARS-CoV-2 positive cohort, hospitalized patients had significantly lower micronutrient levels (*p* < 0.01 for all measured micronutrients) compared to those receiving home care.

Taken together, the studies presented in this Research Topic highlight that lipids are far more than passive structural or energy-storage molecules; they are active regulators of inflammation and key determinants of chronic disease risk. The growing evidence linking lipid metabolism with cardiovascular, metabolic, renal, musculoskeletal, hepatic, and neurological disorders points toward new opportunities for earlier disease detection and more targeted interventions. Future research should focus on translating these mechanistic insights into clinically meaningful biomarkers, nutritional strategies, and therapeutic approaches that can prevent, delay, or even reverse the progression of chronic non-communicable diseases.

